# The causal pie model: an epidemiological method applied to evolutionary biology and ecology

**DOI:** 10.1002/ece3.1074

**Published:** 2014-04-19

**Authors:** Maarten Wensink, Rudi G J Westendorp, Annette Baudisch

**Affiliations:** 1 Max Planck Research Group: Modeling the Evolution of Aging, Max Planck Institute for Demographic ResearchKonrad Zuse Strasse 1, Rostock, 18057, Germany; 2 Leyden Academy on Vitality and Ageing, Poortgebouw LUMCRijnsburgerweg 10, AA Leiden, 2333, The Netherlands; 3 Department of Gerontology and Geriatrics, Leiden University Medical CenterC2-R, PO Box 9600, RC Leiden, 2300, The Netherlands

**Keywords:** Agents of selection, aging, causation, causes of mortality, correlated traits, natural selection, semi-neutral mutations, trade-offs

## Abstract

A general concept for thinking about causality facilitates swift comprehension of results, and the vocabulary that belongs to the concept is instrumental in cross-disciplinary communication. The causal pie model has fulfilled this role in epidemiology and could be of similar value in evolutionary biology and ecology. In the causal pie model, outcomes result from sufficient causes. Each sufficient cause is made up of a “causal pie” of “component causes”. Several different causal pies may exist for the same outcome. If and only if all component causes of a sufficient cause are present, that is, a causal pie is complete, does the outcome occur. The effect of a component cause hence depends on the presence of the other component causes that constitute some causal pie. Because all component causes are equally and fully causative for the outcome, the sum of causes for some outcome exceeds 100%. The causal pie model provides a way of thinking that maps into a number of recurrent themes in evolutionary biology and ecology: It charts when component causes have an effect and are subject to natural selection, and how component causes affect selection on other component causes; which partitions of outcomes with respect to causes are feasible and useful; and how to view the composition of a(n apparently homogeneous) population. The diversity of specific results that is directly understood from the causal pie model is a test for both the validity and the applicability of the model. The causal pie model provides a common language in which results across disciplines can be communicated and serves as a template along which future causal analyses can be made.

## Introduction

Life is all about cause and effect. A general concept for thinking about causality facilitates swift comprehension of results, while the vocabulary that belongs to the concept is instrumental in cross-disciplinary communication. Although in and of itself it does not lead to different results than those obtained with more specific models, a general model provides a quick grasp of the commonalities of seemingly diverse situations. Rothman's “causal pie model” (Rothman 1976, 2012) has fulfilled this role in epidemiology, and we propose that it could be of similar value in biology, in particular in evolutionary biology and ecology. We introduce the model, discuss some generalities that derive from it, and show a wide-ranging sample of applications, from semi-neutral mutations to agents of selection.

## The Model

To paraphrase Rothman (2012), the lights at home shine because they each have a light bulb, there is wire to the light bulbs, the switches are on, there is a power grid, and there is a power source. Take any of these factors away, and there is no light: The system contains 500% causality, for all five factors are 100% causative for the shining of the light. There is no limit to the sum of causes for some outcome (Rothman 2012).

**Figure 1:**
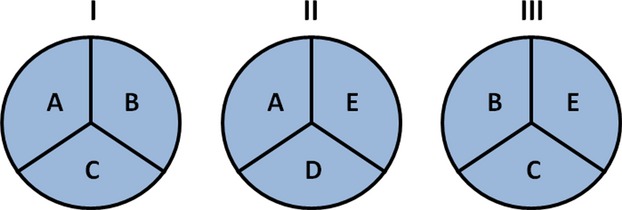
The causal pie model. *Component causes* A–E add up to *sufficient causes* I–III. Every sufficient cause consists of different component causes. If and only if all the component causes that constitute the *causal pie* of a sufficient cause are present, does the sufficient cause exist and does the outcome occur. Hence, the effect of a component cause depends on the presence of its *complementary component causes*, that is, its *complementary set*. I, II, and III can be sufficient causes for the same outcome, or for different outcomes, in which case the outcomes are correlated through the component causes. Examples are given throughout the text.

The causal pie model, depicted in Figure 1 after Rothman's original (1976), represents this way of conceptualizing causality. A *sufficient cause* is a constellation of *component causes*, the *causal pie*, that leads to an outcome. A component cause can be a component of more than one sufficient cause. If and only if all the component causes that make up a causal pie of some sufficient cause are present does the outcome occur. As a result, the effect of a component cause depends on the presence versus absence of the other component causes that make up some causal pie. These are called *complementary component causes*, which jointly make up the *complementary set* of a component cause. In the absence of any one of the complementary component causes, a component cause in itself has no effect. Referring to the example above, if there is no connection to the power grid, varying the position of the switch does not alter the light being on or off.

This model is deterministic in the sufficient causes: If all component causes of some causal pie are present, then the sufficient cause is present and the outcome occurs. This does not prohibit the model from having statistical properties: If one (or more) of the component causes is described statistically, the (joint) distribution function of the (mixed) statistical model describes the outcome as a random variable.

The level of detail required for an analysis determines the biologic outcome of interest, the number of sufficient causes that can exist for the same biologic outcome, and what component causes make up the sufficient causes. For instance, a stroke that is caused by high blood pressure may be classified as an outcome different from a stroke that is not caused by high blood pressure. The latter clearly does not include sufficient causes that contain high blood pressure as a component cause, while the outcome “any stroke” would.

As discussed above, causality adds up to more than 100%. Variation in the occurrence of an outcome due to variation in a specified component cause is bound by 100%. To illustrate this, consider another example (Rothman 2012): The disease phenylketonuria (PKU) is characterized by abnormal metabolism of an amino acid, leading to severe symptoms such as mental retardation. PKU occurs only in people with a mutated gene. The various human diets on planet Earth include the amino acid, so that 100% of the occurrence versus nonoccurrence of PKU on planet Earth is explained genetically. However, consider a hypothetical “other planet”, the inhabitants of which all have the genetic mutation, but do not all have a diet that contains the amino acid. On the other planet, 100% of the observed variation in the occurrence of PKU is explained by variation in the diet.

Variation explained is a matter of prevalence of component causes given some context. Although the percentage of variation explained might be of interest, of even greater interest are statements that are generally true, not just on planet Earth or on the other planet. Such a statement is that 100% of the incident cases of PKU is caused by a mutant gene and 100% is caused by diet. On planet Earth, variation in the prevalence of PKU is explained by genes. Yet, it is another component cause, diet, that is targeted as a treatment. This illustrates why general insights into causality are the more interesting kind of insights.

## Application of the Model to (Evolutionary) Biology and Ecology

The causal pie model has a number of implications for (evolutionary) biology and ecology: It charts under what circumstances component causes have an effect and therefore can be subject to natural selection, and how component causes modulate the effect of and natural selection on other component causes; it shows which partitions of outcomes are useful and which are not; and it provides a way of viewing the composition of a(n apparently homogeneous) population. Each of these points is developed below, and examples are given and framed in terms of the causal pie model.

### Component causes, their effects, and the force of selection

The causal pie model explains when and why biologic factors (component causes) have an effect. When component causes produce an effect, they could affect Darwinian fitness and hence become subject to natural selection. Component causes also influence whether *other* component causes have an effect and become subject to natural selection. We outline some generalities regarding this thinking, and give examples, which are framed in terms of the causal pie model.

**Figure 2:**
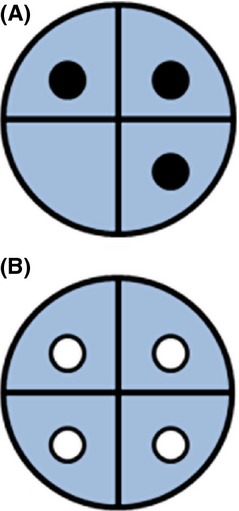
Natural selection in the causal pie model. Selection depends on the effect of presence versus absence of the component cause. (A) If one of the component causes is absent, the other component causes have no effect (barring effects through other causal pies) and are not subject to natural selection, as indicated by the dots being black. (B) If all the component causes are present, the outcome occurs, and all the component causes are subject to the force of selection on the outcome, because without any one of them, the outcome would not occur. If the component causes are A–D and the force of selection on the outcome is S_Ω_, then the force of selection on every component cause, S_X_, equals S_Ω_: S_A_ = S_B_ = S_C_ = S_D_ = S_Ω_.

The presence of all component causes of a causal pie constitutes a sufficient cause, which implies that the outcome occurs. Each of the component causes is then subject to the full force of natural selection on the outcome, for without any one of them, the outcome would not occur (Fig. 2). The force of selection on the outcome is not partitioned over the component causes. Just like the sum of causes exceeds 100%, the force of selection on causes exceeds the force of selection on outcomes. Some component causes will not be determined intrinsically, such as the environment. These component causes will not be subject to natural selection. Still, it will usually be possible to define an intrinsic component cause that is subject to natural selection, such as avoidance of some environmental factor(s).

In the absence of any one of its complementary component causes, the emergence of a component cause has no effect and therefore is not subject to natural selection (barring effects through other sufficient causes). However, component causes are part of the complementary sets of all the other component causes in a causal pie. A component cause that does not give rise to an outcome immediately does change the set of component causes against which yet other component causes arise (e.g., new mutations). It thus affects the chance that another component cause will arise in the context of its complementary set and hence make the outcome occur. In the long term, this gets picked up by evolution. Below, we give a wide-ranging sample of applications of these principles.

#### Example 1: Agents of selection

Wade and Kalisz (1990) recognize that “the fitness of an individual is the result of the interaction of the phenotype with the environment and not an intrinsic feature of either one”, the phenotype being caused by some gene of interest (Wade and Kalisz 1990, pg. 1949). In this context, they discuss that the selection of a gene that gives a fitness (dis)advantage in a particular environment depends on whether that environment is indeed present. They call the environment the “causal agent of selection” of the gene, as the environment causes the gene to be selected.

Wade and Kalisz's realization is a special case of the general principle that selection on a component cause depends on the presence or absence of its complementary set. The effect of the component cause “gene” depends on its complementary component cause “environment”. The complementary set can certainly be environmental, but can be essentially anything that completes a causal pie. If the interaction of many different genes is necessary to bring about some effect, as in the next example, genes act as each other's complementary component causes.

#### Example 2: Semi-neutral mutations

Genetic mutations can be semi-neutral: While they do not have an immediate phenotypic effect, they pave the way for non-neutral change over evolutionary time (Wagner 2011). This happens when a number of mutations have to be acquired before causing phenotypic change. The order of occurrence and the way in which the mutations are generated may be completely random. Only the last mutation seems non-neutral and causes a change in the phenotype, but it is facilitated by the earlier, semi-neutral, mutations. The earlier changes are semi-neutral, because they seem neutral at first sight, but eventually get picked up by evolution.

In terms of the causal pie model, new mutations arise in the context of the existing mutations, which determine whether the complementary set is present. The first few mutations are insufficient to have a phenotypic effect, but they do change the context against which new mutations arise. At one point, the new mutation arises in the presence of the other mutations necessary for phenotypic change, that is, its complementary set of a sufficient cause for phenotypic change. All of the mutations are equally causal to the outcome. Yet, all mutations before the one that leads to phenotypic change seem neutral, because their complementary set for phenotypic change is lacking.

#### Example 3: selection for versus selection of

Selection *of* refers to an empirical observation of what is selected, while selection *for* means that there is selection due to a causal effect of some trait on vital rates (Sober 1984; discussed in Endler 1986). These sorts of selection differ if traits are correlated, which is a well-established area of research (Lande 1982; Lande and Arnold 1983; Arnold and Wade 1984; Mitchell-Olds and Shaw 1987). As an example, consider a population of mice in which small mice have a selective advantage, for instance because they are not seen by predators. Suppose also that the same gene that controls body size causes large mice to have blue eyes, while it causes small mice to have green eyes. Finally, suppose that having blue eyes versus green eyes by itself does not affect mortality and fecundity. In this example, there is selection *for* small body size, because this has a direct effect on the risk of predation. There is selection *of* not only small body size, but also of green eye color. Although the latter has no effect on fitness, selection results from the correlation of eye color with body size.

In the causal pie model, traits are correlated when they share one or more component cause(s). For instance, in Figure 1, A is a component cause for both sufficient causes I and II, leading to correlation. If one sufficient cause is subject to natural selection, the force of selection extends to all component causes. Some of these component causes can be component causes also of sufficient causes for neutral traits, leading to selection *of*. In Figure 1, selection on sufficient causes I and II leads to selection on component causes A–E. Sufficient cause III is not selected for (it is in itself a neutral trait). Still, there is selection of sufficient cause III, because there exists selection on all its component causes (B, C, E).

#### Example 4: Trade-offs

Correlated traits of particular interest are trade-offs (e.g., Stearns 1989; Stearns 1992). For instance, an organism could have to balance the risk of starvation against the risk of being eaten by a predator while foraging. The preferred choice under this trade-off obviously depends on the presence of food versus the presence of predators. To give an example at a molecular level, consider the case in which a higher metabolic rate leads to higher short-term survival, for instance by higher vigilance, but also leads to increased damage accumulation due to oxygen radicals. This trade-off depends on the presence of scavengers for oxygen radicals and on the vulnerability of the macromolecules to oxygen radicals.

A component cause has an effect only in the presence of its complementary set. This is true also for component causes that give rise to trade-offs. Therefore, trade-offs depend on the presence or absence of complementary sets. In the first example above, predators and food (complementary component causes) may be distributed probabilistically over the habitat or over time. As the causal pie model is also probabilistic, it fits the examples above well. The probability distribution of either outcome depends on the probability distributions of the complementary component causes, which determine the preferred behavior.

Trade-offs depend on the presence of complementary component causes. There is selection toward optimal outcomes given the complementary component causes that are present: Evolution will have the tendency to optimize within the limitations of the trade-off. For instance, in the molecular example in the first paragraph of this section, the trade-offs depend on the presence of scavengers for oxygen radicals and on the vulnerability of the macromolecules to oxygen radicals. Given the vulnerability of macromolecules to oxygen radicals, and given a certain concentration of scavengers, evolution will optimize metabolism. At the same time, there is selection on the complementary component causes: Evolution will have the tendency to push the boundaries of the trade-off, such that the trade-off becomes less restrictive. To stay with the example of oxidative damage, evolution will tend toward using less vulnerable macromolecules (if possible), and toward increasing scavenging capacity, thus changing the trade-off that it faces.

#### Example 5: The problem with age-specific genes

The evolutionary theory of aging is sometimes defined in terms of “age-specific genes”, genes that are active at a particular age or during a particular age range and not before or after. The idea is that natural selection acts less forcefully against detrimental genes the later they are expressed (Medawar 1952; Williams 1957; Hamilton 1966; Charlesworth 1994). However, age as such causes nothing and cannot activate or deactivate genes (Kirkwood 1977; Kirkwood and Shanley 2010; Wensink 2013). At first sight, a way around this objection is to define a “substance S” (for senescence, deterioration with age), that is not in itself “deleterious in any normal sense” (Dawkins 2006), in line with Williams's genes that “act differently in a different somatic environment” (Williams 1957). Some independent somatic change would trigger the expression of deleterious genes at high ages, thus making them “age-specific”.

The causal pie model shows that it is not logically correct to say that substance S is not deleterious “in any normal sense”. The causal pie is made up of two component causes – a certain concentration of substance S, and a substance S-sensitive gene – which together form a sufficient cause for deterioration and death. Completing the causal pie, substance S is as causal to the detrimental effect as is the substance S-sensitive gene. Without substance S, there would be no senescence, just like without the substance S-sensitive gene, there would be no senescence. As a result, the force of natural selection on deterioration that results from the expression of the deleterious gene extends to both the gene and substance S (Wensink 2013). Theories about age-specific genes are in fact theories about state-specific genes and some state variable, in this case, the concentration of substance S. Both are part of the evolutionary analysis.

### Partitioning of outcomes

Generally speaking, partitions are useful only when they are based on qualities that are mutually exclusive. If this is not the case, something could belong to more than one partition. Because the whole point of partitioning is to divide things up, making partitions based on qualities that are not mutually exclusive should not be attempted, because it will fail.

In the causal pie model, multiple component causes need to interact for an outcome to occur. It is not useful to partition outcomes as caused by one versus the other component cause, as an outcome could be caused by both component causes at the same time. It can be useful, however, to make a partition between outcomes in which a particular component cause plays a role, versus outcomes in which it does not.

#### Example: Extrinsic mortality

Although they acknowledge a role for vulnerability of an organism to extrinsic threats, Carnes et al. (2006) set out to partition mortality “based on whether the primary cause of death does or does not originate from within the organism”. Extrinsic mortality has been invoked at many points in evolution (e.g., Medawar 1952; Williams 1957; Kirkwood 1977; but see Caswell 2007).

Mortality, however, is a prime example in biology of interacting component causes, some extrinsic, some intrinsic to the organism. Therefore, it is not possible to characterize mortality either extrinsic or intrinsic. A typical example is predation, where the presence of a predator (component cause 1) interacts with a state of vulnerability of the prey (component cause 2) and an environment that co-determines if prey and predator effectively meet, for instance a quality of vegetation (component cause 3). These three component causes could be partitioned in more detailed component causes if required, such as the cardiopulmonary capacity or fur color of predator and prey.

*Component causes* of mortality can be partitioned in extrinsic and intrinsic, but *mortality itself* cannot, for the simple reason that extrinsic and intrinsic mortality are not mutually exclusive. Acknowledging a role for vulnerability in “extrinsic mortality” does not take away this objection. None of the component causes is more causative to the outcome, as without any one of them, the outcome would not occur. One could propose that there can be no predation without a predator and that therefore the (extrinsic) presence of a predator must be the most causative cause for death by predation. However, neither is there predation without a prey. Being prey is something “intrinsic”. There is no such thing as “most causative cause”. A partitioning that could have some utility is a partition of mortality in which an extrinsic component cause plays a role, versus mortality in which it does not. The term “extrinsic mortality” retains some meaning in this respect, although one could wonder whether purely intrinsic mortality exists at all.

### Population heterogeneity

Populations are heterogeneous, and an observation at the population level may be quite different from individual-level processes (Vaupel and Yashin 1985). A population may seem homogeneous, but below the surface, organism may differ greatly. Any change that may happen may have differential effects on each organism.

A way to look at heterogeneity in a population is to view all members of the population as different collections of causal pies that are sufficient causes for some outcome, for instance death. Some of those members will have many causal pies that are almost “filled in”, that is, most component causes that make up the causal pie are present. Others will have mostly “empty” causal pies. Yet, others will have roughly equally filled causal pies, but the causal pies will be made up of different component causes. These individuals will be vulnerable to some types of stress, but robust to others, differing from individual to individual. How newly imposed stress affects each member of the population depends on the component causes that are yet present.

#### Example: Frailty along the life course

**Figure 3:**
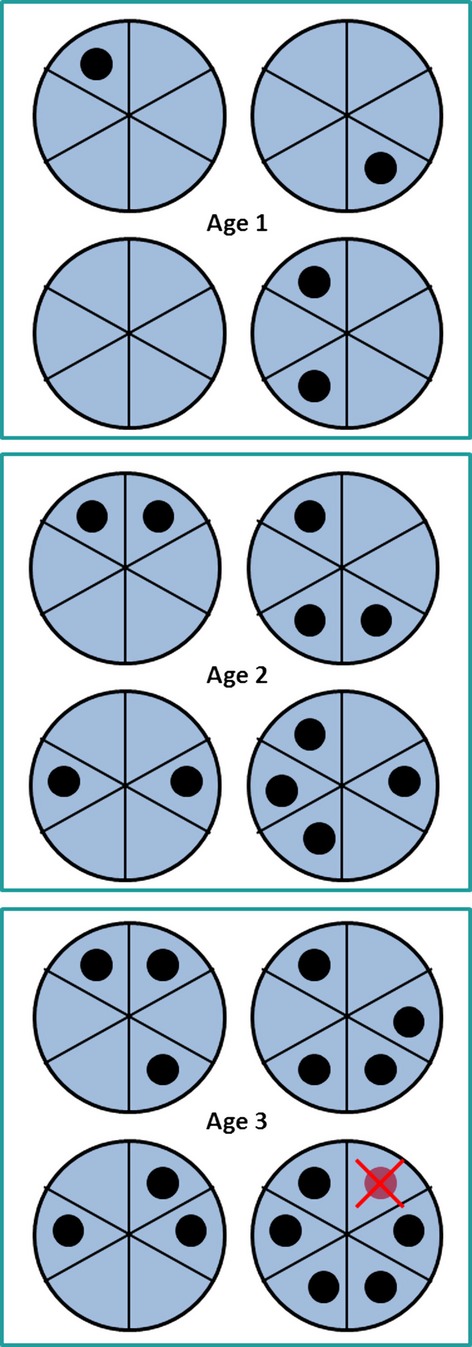
Frailty along the life course. An organism has a number of sufficient causes for mortality, that is, causal pies. A black dot in a part of a pie means that the component cause is present. Along the life course, more and more component causes become present, but the organism seems healthy when mortality and fecundity are considered. Yet, fewer and fewer additional damages are necessary to lead to death: The organism gets frailer. When the last component cause of some causal pie emerges, indicated by the cross at age 3, the deterioration becomes clearly manifest and the organism dies.

A physiological view of human aging proposed by Izaks and Westendorp (2003) is that there exist multiple causal pies that form sufficient causes for deterioration and death which are slowly and steadily being filled in over the lifetime of an individual. Even apparently healthy older individuals are more vulnerable, because they have accumulated damages that in and of themselves do not cause disease or mortality, but that are part of causal pies that steadily have more component causes present. With more component causes already present, even if organisms appear healthy, it is easier to acquire the remaining component causes, leading to disability and death (Fig. 3).

This view is by no means limited to humans, but applies to all living organisms. Vital rates may be improving during part of the life cycle, but causal pies of disability and death are being filled in concurrently, making the distance to disability and death shorter. While physiological markers of aging are already deteriorating, that is, physiological aging, they may not yet lead to deterioration of mortality and fecundity, that is, demographic aging. Yet, any additional stress will have a much larger chance of completing a causal pie in older individuals, leading to quick deterioration of vital rates later in life. This explains why demographic aging can trail physiological markers (component causes) with a delay and why apparently healthy organisms may differ greatly in their vulnerability to stress. Together with trade-offs, causal pie models of aging may also contribute to explain the recently revealed diversity of aging patterns across the tree of life (Jones et al. 2013).

## Discussion

All the results discussed in this paper derive from one and the same principle: the causal pie model. Still, any and all of these results can be derived without reference to the causal pie model. For instance, no evolutionary biologist would be surprised to learn that if genetic variation in some respect is initially subject only to random drift, it can suddenly become subject to natural selection if the environment changes such that the genetic variation becomes relevant to Darwinian fitness. This is standard evolutionary theory; no causal pie model is needed to explain it. Then what is the utility of the causal pie model, in particular when compared to other models of causation?

In reality, science is subject to significant uncertainty. In a given natural system, not all causal pies may be known or identifiable, or the presence versus absence of (some of) the component causes may be ascertained only at a statistical level. More involved models, such as structural equation modeling (Pearl 2009), are then necessary to discover the underlying causal relationships. Causation is then expressed in probabilistic terms, such that A causes B if the probability of B in the presence of A is greater than in the absence of A, that is, P(B|A) > P(B|¬A). As pointed out in section “The model”, even though it is not itself a statistical model, the causal pie model is fully compatible with this probabilistic representation of causal relationships. The probability of the presence of all component causes of a pie chart is the product of the probabilities of the presence of each individual component cause, such that if A–D are all the component causes of causal pie I, it holds that P(I) = P(A)P(B)P(C)P(D). If there are more than one sufficient causes I–III (causal pies) for outcome Ω, it holds that P(Ω) = P(I) + P(II) + P(III).

To reveal underlying causal relations in a data set, the causal pie model offers no alternative to the appropriate tools of data analysis, such as structural equation modeling [Shipley (2000) and Grace (2006) are helpful references for such modeling in biology] or quantitative methods of natural selection (e.g., Lande 1982). However, we do not propose the causal pie model to take the place of these methods. Rather, it should be seen as a helpful tool of conceptualizing causation whenever this simplest model suffices. The causal pie model is a very simple model, perhaps the simplest, that captures the basic workings of causation. The model is instrumental in understanding a range of results, such as those discussed in this paper, and in avoiding common mistakes, such as partitions between nonmutually exclusive component causes and summing causes to 100%. In the meantime, it contains little jargon and no mathematics, so that the model is easily and intuitively accessible.

Although we use different words in various disciplines, we describe the same phenomena. The causal pie model can help to make bridges, so that various disciplines can draw on and be inspired by each other's finding. The range of examples that we give is a good demonstration of the utility of the causal pie model in this respect. We propose that the causal pie model provides an effective framework for thinking about causation, that it helps to avoid mistakes, and that it provides a simple common language in which results can be communicated across disciplines.
